# β-secretase inhibition prevents structural spine plasticity deficits in *App*^*NL-G-F*^ mice

**DOI:** 10.3389/fnagi.2022.909586

**Published:** 2022-07-22

**Authors:** Tanja Blume, Severin Filser, Carmelo Sgobio, Finn Peters, Ulf Neumann, Derya Shimshek, Takashi Saito, Takaomi C. Saido, Matthias Brendel, Jochen Herms

**Affiliations:** ^1^German Center for Neurodegenerative Diseases, Munich, Germany; ^2^Center for Neuropathology and Prion Research, Ludwig-Maximilians-University of Munich, Munich, Germany; ^3^Institute for Stroke and Dementia Research, University Hospital, Ludwig-Maximilians-University of Munich, Munich, Germany; ^4^Evotec SE, Hamburg, Germany; ^5^Department of Neuroscience, Novartis Institutes for BioMedical Research, Basel, Switzerland; ^6^Laboratory for Proteolytic Neuroscience, RIKEN Center for Brain Science, Saitama, Japan; ^7^Department of Neurocognitive Science, Institute of Brain Science, Nagoya City University Graduate School of Medical Sciences, Aichi, Japan; ^8^Munich Cluster for Systems Neurology, Munich, Germany; ^9^Department of Nuclear Medicine, University Hospital of Munich, Ludwig-Maximilians-University of Munich, Munich, Germany

**Keywords:** Alzheimer's disease, dendritic spines, *in vivo* two-photon microscopy, BACE1-inhibition, APP knock-in, *App* ^*NL-G-F*^

## Abstract

All clinical BACE1-inhibitor trials for the treatment of Alzheimer's Disease (AD) have failed due to insufficient efficacy or side effects like worsening of cognitive symptoms. However, the scientific evidence to date suggests that BACE1-inhibition could be an effective preventative measure if applied prior to the accumulation of amyloid-beta (Aβ)-peptide and resultant impairment of synaptic function. Preclinical studies have associated BACE1-inhibition-induced cognitive deficits with decreased dendritic spine density. Therefore, we investigated dose-dependent effects of BACE1-inhibition on hippocampal dendritic spine dynamics in an APP knock-in mouse line for the first time. We conducted *in vivo* two-photon microscopy in the *stratum oriens layer of* hippocampal CA1 neurons in 3.5-month-old *App*^*NL-G-F*^GFP-M mice over 6 weeks to monitor the effect of potential preventive treatment with a high and low dose of the BACE1-inhibitor NB-360 on dendritic spine dynamics. Structural spine plasticity was severely impaired in untreated *App*^*NL-G-F*^GFP-M mice, although spines were not yet showing signs of degeneration. Prolonged high-dose BACE1-inhibition significantly enhanced spine formation, improving spine dynamics in the AD mouse model. We conclude that in an early AD stage characterized by low Aβ-accumulation and no irreversible spine loss, BACE1-inhibition could hold the progressive synapse loss and cognitive decline by improving structural spine dynamics.

## Introduction

Beta-site amyloid-precursor-protein cleaving enzyme 1 (BACE1) has for several years been considered a potential drug target for therapeutic intervention in Alzheimer's Disease (AD). In 2018, several BACE1-inhibitors were evaluated in phase-III clinical trials, but these trials were either terminated prematurely, due to side effects such as cognitive worsening, or the treatment was subsequently found to be insufficiently effective in stopping the disease progression (Piton et al., 2018; Knopman, 2019; Hampel et al., 2020). However, since BACE1-secretase is the rate-determining step in the generation of the amyloid-beta (Aβ)-peptide, the therapeutic potential of BACE1-inhibition is still being investigated (Hampel et al., 2020).

Previous *in vivo* imaging studies in mice have revealed that BACE1-inhibitor treatment causes a persistent reduction in the formation of new dendritic spines as well as in the total spine density in wild-type mice (Filser et al., 2015; Blume et al., 2018), mainly due to the disrupted processing of the BACE substrate seizure-related gene6 protein (Sez6) (Zhu et al., 2016, 2018). These preclinical observations provide an explanation for the deterioration in cognitive function observed during clinical trials with BACE1-inhibitors, as the structural dynamics of dendritic spines represent a central cellular mechanism in memory formation (Chklovskii et al., 2004; Holtmaat and Svoboda, 2009; Xu et al., 2009; Yang et al., 2009).

Interestingly, it was also shown that the effect of BACE1-inhibition on the plasticity of dendritic spines is dose-dependent in wild-type mice. While high BACE1-inhibitor concentrations lead to a decrease in spine plasticity, such detrimental effects were not observed at lower doses (Filser et al., 2015).

Since physiological Aβ-levels like in wild-type conditions have been shown to be necessary for neuronal survival and beneficial for synaptic plasticity, whereas high concentrations of Aβ-oligomers, a hallmark of AD pathology, were proven to be synaptotoxic (Hardy and Selkoe, 2002; Giedraitis et al., 2007; Haass and Selkoe, 2007; Puzzo et al., 2008), we hypothesize that the effect on dendritic spine dynamics would differ between wild-type mice and AD mouse model mice. Furthermore, the concentration of BACE1 and its enzymatic activity are two-fold elevated in AD as compared to healthy controls (Yang et al., 2003).

Based on the failure of BACE1-inhibitor trials, it is assumed that BACE1-inhibition therapy may not be able to improve cognition in symptomatic patients because the progressive and irreversible decline of synapses and neurons has already been taken place. Thus, BACE1-inhibitor intervention might be ideally started before to the accumulation of the Aβ-protein, synaptic loss as well as the onset of potentially irreversible cognitive deficits in order to achieve a positive outcome on cognition (Moussa-Pacha et al., 2019; Hampel et al., 2020).

Since the hippocampus is among the earliest brain areas to be affected in AD (Braak and Braak, 1991) and is a key brain region in episodic learning and memory consolidation (McClelland et al., 1995), we assessed the impact of primary-preventive BACE1-inhibition on hippocampal dendritic spine dynamics in an amyloid mouse model. Therefore, we performed longitudinal *in vivo* two-photon imaging of basal dendrites in the hippocampal CA1 *stratum oriens* layer distant to Aβ-deposits in groups of young *App*^*NL*−*G*−*F*^- mice treated for 4 weeks with a high- and a low-dose of a potent and selective BACE1-inhibitor (NB-360) or vehicle (Neumann et al., 2015, 2019). The advantage of the *App*^*NL*−*G*−*F*^ mouse model is that this knock-in line does not overexpress APP like transgenic AD mouse models (Saito et al., 2014). Groups of wild-type animals (*App*^*wt*^GFP-M) were likewise treated with both BACE1-inhibitor doses or vehicle to examine dose-dependent effects of BACE1-inhibition on physiological spine dynamics in the hippocampus, and to compare our observations with previous studies conducted in the somatosensory cortex (Filser et al., 2015; Zhu et al., 2016; Blume et al., 2018).

## Materials and methods

### BACE1-inhibitor

The BACE1-inhibitor NB-360 was synthesized and kindly provided by Novartis Pharma AG. The pharmacological properties of NB-360 have been reported previously (Neumann et al., 2015, 2019). Mice aged 4–5 months were fed *ad libitum* with food pellets formulated with NB-360 at a high-dose (0.29 g/kg), a low-dose (0.05 g/kg), or control pellets for four consecutive weeks.

### Animals

Male heterozygous GFP-M mice [Tg(Thy1-EGFP)MJrs from Jackson Laboratory, JAX stock #007788] (Feng et al., 2000) were crossed with the knock-in mouse line *App*^*NL*−*G*−*F*^ (Saito et al., 2014) to generate the line *App*^*NL*−*G*−*F*^GFP-M. *App*^*NL*−*G*−*F*^ mice carry a mutant APP gene encoding the humanized Aβ-sequence with the Swedish, Beyreuther/Iberian and Arctic mutation. To establish a wild-type line we backcrossed *App*^*NL*−*G*−*F*^ mice with C57BL/6J mice (Jackson Laboratory, JAX stock #000664) for five generations and these progeny were crossed with GFP-M mouse line (*App*^*wt*^GFP-M or wild type). Mice were group-housed under pathogen-free conditions and bred in the animal housing facility at the German Center for Neurodegenerative Diseases, with water and mouse chow provided *ad libitum* until the start of NB-360 treatment as described above. The animals were kept in a dedicated facility at 21 ± 1°C with a 12/12-h light/dark cycle. Mice were housed separately after implantation of the hippocampal window, as described below. All animal procedures followed a protocol approved by the local authorities (Regierung von Oberbayern, TVA-AZ: 55.2-1-54-2532-214-2016).

### IHC and confocal imaging

Brain sections, sliced with a VT1000S Vibratom (Leica Microsystems, Wetzlar, Germany) of *App*^*NL*−*G*−*F*^GFP-M mice, were imaged with the LSM-780 confocal microscope (Zeiss, Oberkochen, Germany) using a 40/1.4× oil objective (Zeiss). Three regions of interest (ROIs) were chosen per mouse in the hippocampal CA1 *stratum oriens* layer for the acquisition of three-dimensional overview z-stacks measuring 216 × 216 × 90 μm with a resolution of 0.423 × 0.423 × 1.00 μm in the X-, Y- und Z-planes. Brain sections were stained with anti-eGFP (A-21 311, Invitrogen, Waltham, Massachusetts, USA) to enhance the intrinsic eGFP signal (488 nm) and seven to ten dendrites were selected proximal to (<50 μm) and distant to (>50 μm) Aβ-deposits, stained with NAB228 (647 nm; sc-32 277, Santa Cruz, California, USA) and FSB (405 nm; CAS 891 180 931, Merck, Burlington, Massachusetts, USA). We then acquired high-resolution images (0.104 × 0.104 × 0.395 μm in X-, Y- und Z-resolution) of the selected dendrites.

### Hippocampal window implantation

A hippocampal window was implanted as previously reported (Gu et al., 2014). In short, mice were anesthetized by intraperitoneal injection of ketamine/xylazine mixture (13.0/1.0 μg/g body weight). Rimadyl (7.5 μg/g) and baytril (2.5 μg/g) were administered for analgesic and antibiotic treatment, respectively.

The target region of the window above the dorsal hippocampus (anteroposterior: −2.2 mm; mediolateral: +1.8 mm relative to bregma) was thinned out with a dental drill until the bone could be removed. A sterile, blunt tip attached to a vacuum pump was used to aspirate the neocortex as well as the *corpus callosum* above the hippocampus. To gain optical access to the dorsal hippocampus, a sterile custom-made metal tube (diameter 3 mm; height, 1.5 mm), sealed at one end with a circular glass coverslip, was inserted into the trepanation. A small metal bar was aligned parallel to the surface of the window and fixed in place with cement to ensure consistent repositioning of the mouse during subsequent imaging sessions. *In vivo* two-photon was started 4 weeks after the hippocampal window preparation to allow complete recovery from surgery.

### *In vivo* two-photon imaging

During the 1-h imaging sessions, anesthesia was maintained with the use of low-dose isoflurane (1%). For two-photon imaging, we used a LSM-880 laser scanning microscope (Zeiss) using a CFI LWD Plan Fluorite 16×/0.8 DIC immersion objective (Nikon, Chiyoda, Tokyo, Japan) and a coupled Ti:Sapphire Laser Chameleon Vision S (Coherent, Santa Clara, USA). eGFP was excited at a wavelength of 920 nm. In total, three ROIs were chosen for each mouse and three-dimensional overview z-stacks of 209 × 209 × 180 μm with 0.205 × 0.205 × 1.00 μm in X-, Y- und Z-resolution were acquired. In order to identify the origin of the basal dendrites in the *stratum oriens* of the CA1-region as well as the order of branching, the somata of the corresponding pyramidal cells were also recorded in these overview images. Within the overview images, single oblique dendrites of the second order of branching and running parallel to the brain surface were selected for high-resolution imaging (0.082 × 0.082 × 1.00 μm X-, Y- und Z-resolution with a 15× optical zoom). Knowing the coordinates of the overview images and the specific arrangement of the cell bodies of the eGFP expressing neurons, allowed us to a precisely realignment the same imaging volume including the selected dendrites over a period of 6 weeks.

### Analysis of *in vivo* and *ex vivo* data

The spine density *in vivo* was determined similarly as described before (Holtmaat et al., 2005; Fuhrmann et al., 2007; Holtmaat and Svoboda, 2009). In short, *in vivo* images were analyzed using the ZEN Software Black Edition (Zeiss). In each animal, seven to ten dendritic segments of 20–50 μm in length were analyzed. Along this segment, all distinct, laterally protruding spines with a length > 0.4 μm (Holtmaat and Svoboda, 2009) were scored as dendritic spines, regardless of shape. Spines were monitored at all following time points. A spine was considered the same if it did not change its position along the dendrite by >0.5 μm between two imaging time points. Lost spines with <0.4 μm length were labeled in red. Gained spines with >0.4 μm length were labeled in green. Due to the relatively limited resolution in z-direction, only spines emanating from the dendrite in the x-y direction were counted.

*Ex vivo* image analysis was also performed using ZEN software. In each animal, seven to ten dendrites of 20–50 μm length were analyzed, whereby all laterally emanating spines > 0.4 μm in length were counted manually, with mushroom-shaped, stubby and thin spines being distinguished according to established criteria (Holtmaat and Svoboda, 2009).

For illustration purpose only, image stacks were adjusted for contrast and brightness.

### Statistics

Quantifications and statistical analysis were performed using Prism Software 7 (GraphPad Software, San Diego, California, United States). All analyses were performed by an experimenter blinded to the experimental conditions. Data were normally distributed according to Shapiro-Wilk or D'Agostino-Pearson test. For *in vivo* time series data, two-way ANOVA followed by Bonferroni *post-hoc* test were performed to statistically determine significance. If ANOVA treatment main factor was significantly changed, we conducted also one-way ANOVAs followed by Bonferroni *post-hoc* test to obtain the significance between the treatment groups. For assessment of inter-group differences at single time points, Student's *t*-test (unpaired, two-sided) was applied. All results are presented as mean ± SEM. *P*-values < 0.05 are defined as statistically significant.

## Results

### The *App^**NL*-*G*-*F**^* mouse model shows a decrease in mushroom spines but not in total spine density distant to Aβ-deposits

**Figure 1:**
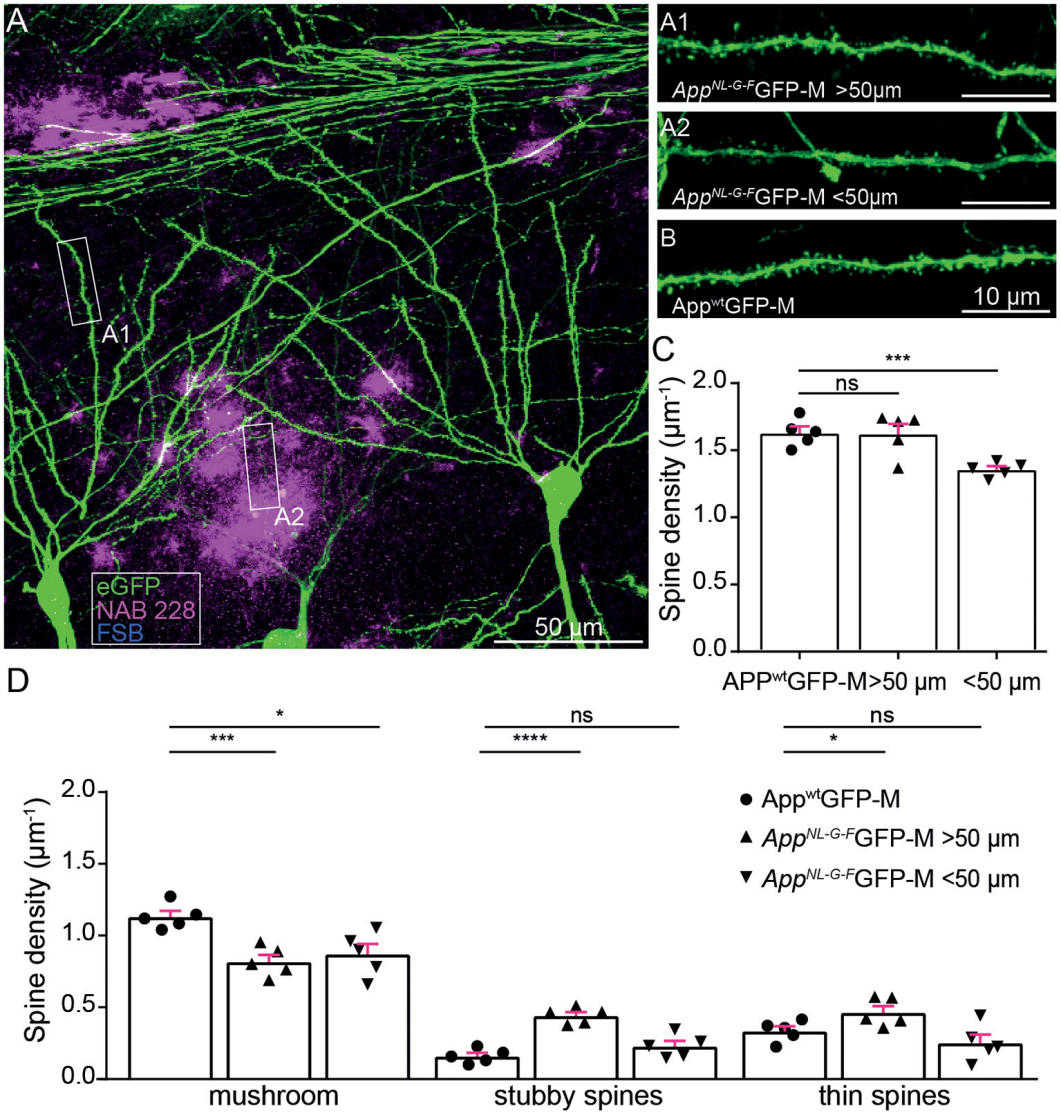
*Ex vivo* spine analysis showed a decrease in mushroom spines but not in total spine density distant to Aβ-depositions in *App*^*NL*−*G*−*F*^ mice. **(A)** The overview picture of a section in the *stratum oriens* CA1 subfield of the hippocampus is shown as a maximum intensity projection. eGFP expression (green), oligomeric Aβ (NAB228, magenta), fibrillar Aβ (FSB, cyan). **(A1,A2)** High-resolution confocal images of second-degree branched dendrites distant (>50 μm) and proximal (<50 μm) to Aβ-depositions of *App*^*NL*−*G*−*F*^GFP-M mice as well as in *App*^*wt*^GFP-M animals **(B)** are shown as maximum intensity projections. **(C,D)** Density of total, mushroom, thin and stubby spines of *App*^*wt*^GFP-M mice and *App*^*NL*−*G*−*F*^GFP-M mice distant (>50 μm) and proximal (<50 μm) to Aβ-depositions. *N* = 5 male mice, *n* = 7–10 dendrites. Data are presented as mean ± SEM. Two-sample Student's *t*-test results: *p < 0.05, ***p < 0.001, ****p < 0.0001.

First, we investigated *ex vivo* whether the APP knock-in mouse model shows reduced hippocampal spine density or altered spine morphology. In particular, we examined dendrites in the *stratum oriens* of the CA1-region in the hippocampus proximal to (<50 μm) and distant to (>50 μm) FSB and NAB228 immunostained Aβ-deposits in 5-month-old *App*^*NL*−*G*−*F*^GFP-M mice (Figures 1A,B). We found a decrease in total spine density proximal to Aβ-deposits [*t*_(8)_ = 5.24; *p* = 0.0008], whereas no spine loss could be observed distant to Aβ-deposits [*t*_(8)_ = 0.07318; *p* = 0.9435; Figure 1C]. A more detailed comparison of spine morphology showed a significant loss of mushroom spines [*t*_(8)_ = 3.274; *p* = 0.0113; Figure 1D] as well as a trend toward fewer thin spines proximal to Aβ-depositions in the *App*^*NL*−*G*−*F*^GFP-M mice compared to total mushroom spine density in *App*^*wt*^GFP-M mice. We also observed a change in spine morphology including a significant decrease in mushroom spines density distant to Aβ-deposits [*t*_(8)_ = 5.143; *p* = 0.0009; Figure 1D] in *App*^*NL*−*G*−*F*^GFP-M mice compared to total mushroom spine density in *App*^*wt*^GFP-M mice, although there was no decrease in total spine density (Figure 1C). This can be explained by the simultaneous increase in the density of stable [*t*_(8)_ = 8.493; *p* ≤ 0.0001] and thin spines [*t*_(8)_ = 2.363; *p* = 0.0457] distant to the Aβ-deposits.

Since mushroom spines are of critical importance for long-term memory formation (Bourne and Harris, 2007; Tackenberg et al., 2009), we suggest that a loss of mushroom spines both proximal and distant to Aβ-deposits, causes a deficit in the structural plasticity of dendritic spines.

### Non-plaque-associated hippocampal dendritic spine alterations in *App^**NL*-*G*-*F**^* mice

In order to clarify whether the impaired spine morphology distant to Aβ-plaques in the *App*^*NL*−*G*−*F*^ mouse model (Figure 1D) was caused by altered structural plasticity of dendritic spines, we analyzed spine density and dynamics of dendrites on basal tufts in the *stratum oriens* layer of the CA1-region in the hippocampus distant to Aβ-plaques for six consecutive weeks *in vivo*.

**Figure 2:**
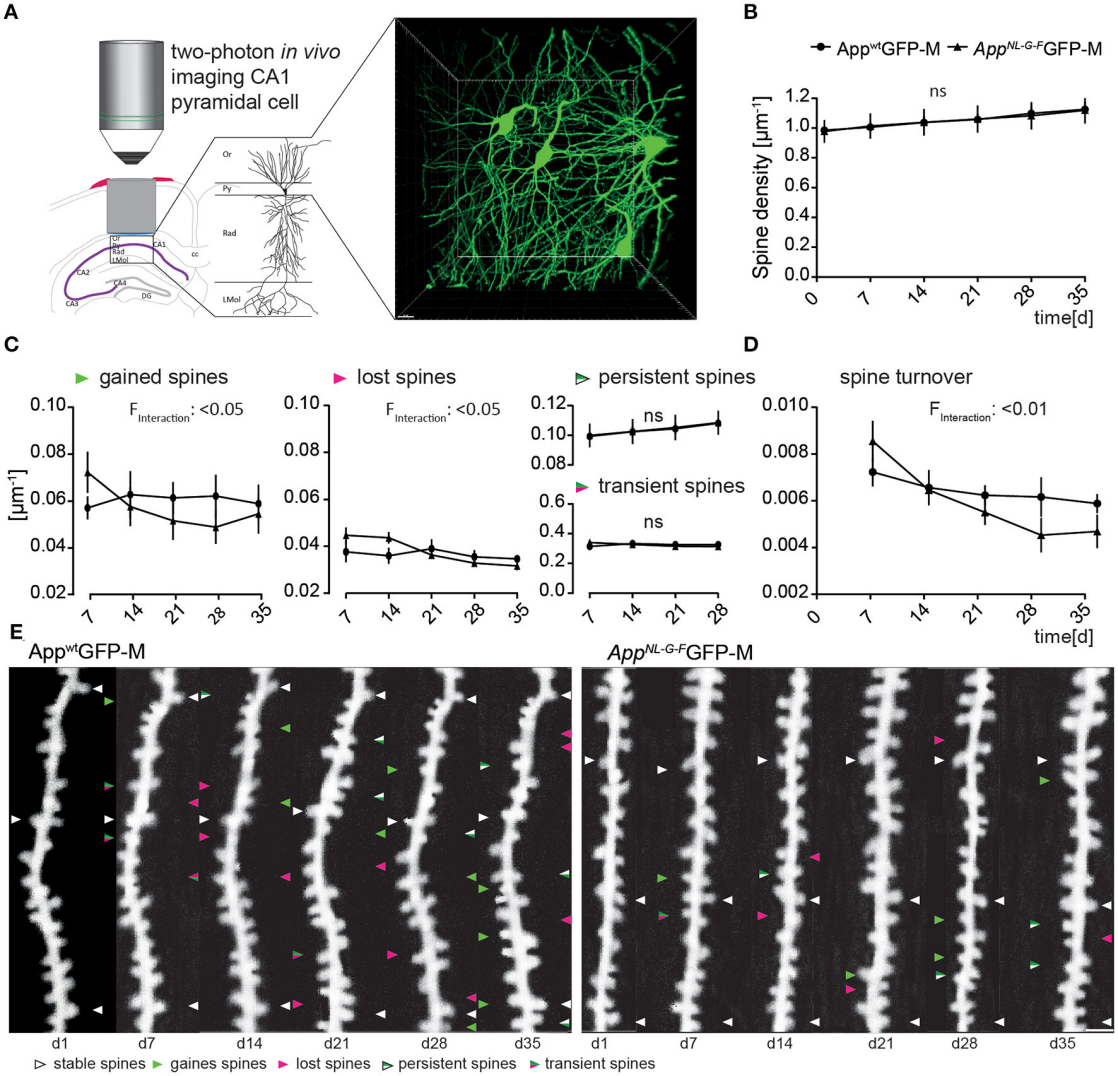
The *App*^*NL*−*G*−*F*^ mouse model showed altered plasticity of dendritic spine dynamics *in vivo* distant to Aβ-deposits. **(A)** Left, schematic illustrating two-photon *in vivo* microscopy of basal dendrites in the *stratum oriens* (Or) of the CA1-region in the hippocampus. Right, maximum intensity projection of a z-stack acquired up to a depth of 180 μm below the *corpus callosum* comprising *stratum oriens* (SO), *stratum pyramidale* (Py) and *stratum radiatum* (Rad). Scale bar: 15 μm. **(B)** The spine density distant to Aβ-deposits in *App*^*NL*−*G*−*F*^GFP-M mice was not significantly changed compared to *App*^*wt*^GFP-M mice (*p* = 0.9753) **(C)** The fractions of gained (*p* = 0.0208) and lost spines (*p* = 0.0238) were significantly decreased in *App*^*NL*−*G*−*F*^GFP-M mice compared to *App*^*wt*^GFP-M animals, while the fractions of persistent (*p* = 0.9593) and transient spines (*p* = 0.9521) did not differ. **(D)** Consequently, the spine turnover ratio was significantly decreased in *App*^*NL*−*G*−*F*^GFP-M mice compared to *App*^*wt*^GFP-M mice (*p* = 0.0064). **(E)** Representative *in vivo* two-photon recordings of eGFP-labeled basal tuft dendrites in the *stratum oriens* of CA1-region in the hippocampus in *App*^*NL*−*G*−*F*^GFP-M and *App*^*wt*^GFP-M mice. Arrowheads mark representative spines that were stable (white), newly formed (green), or lost (magenta). Gained spines that did not stabilize (green/magenta, present < 7 days) were defined as transient, whereas gained spines, that did stabilize were defined as persistent (green/white, present > 7 days). Scale bar: 2 μm. *N* = 6 animals per group, *n* = 7–10 dendrites per animal. Data are presented as mean ± SEM. Bonferroni *post-hoc* test results from two-way ANOVA.

We obtained overview images of the pyramidal cells through the layer's *stratum oriens* to *stratum radiatum* to ensure to image specifically the second branch of basal dendrites of the *stratum oriens* (Figure 2A). Newly appearing and disappearing spines relative to the previous imaging session were marked as “gained” or “lost”, respectively (Figure 2E). In addition, we differentiated gained spines as being persistent or transient spines, according to their lifespan (Figure 2E).

The total spine density did not differ between *App*^*NL*−*G*−*F*^GFP-M and *App*^*wt*^GFP-M mice over the imaging period of 6 weeks (Figure 2B).

However, there was a significant difference in the spine dynamics in *App*^*NL*−*G*−*F*^GFP-M compared to *App*^*wt*^GFP-M animals (Figure 2C). The fraction of newly gained spines was significantly lower [*F*_(4, 40)_ = 3.266; *p* = 0.0208] in *App*^*NL*−*G*−*F*^GFP-M mice, with ~24% fewer spines formed at the last imaging day. The fraction of lost spines was also significantly lower [*F*_(4, 40)_ = 3.164; *p* = 0.0238] in the AD model, but there was no significant difference in the fractions of transient [*F*_(1, 10)_ = 0.0038; *p* = 0.9521] or persistent [*F*_(1, 10)_ = 0.0027; *p* = 0.9593] spines in *App*^*NL*−*G*−*F*^GFP-M compared to *App*^*wt*^GFP-M mice. The difference in spine dynamics lead to a significantly lower spine turnover rate in *App*^*NL*−*G*−*F*^GFP-M compared to *App*^*wt*^GFP-M mice [*F*_(4, 40)_ = 4.182; *p* = 0.0064; Figure 2D]. By the last imaging day, the spine turnover rate of *App*^*NL*−*G*−*F*^GFP-M mice had decreased by 45%, whereas there was only a slight decrease of 14% in *App*^*wt*^GFP-M animals.

### BACE1-inhibitor treatment in high dose rescues dendritic spine formation in *App^**NL*-*G*-*F**^* mice

Next, it was assessed whether early intervention with the BACE1-inhibitor NB-360 could rescue the impaired structural spine plasticity in the *App*^*NL*−*G*−*F*^ mouse model.

**Figure 3:**
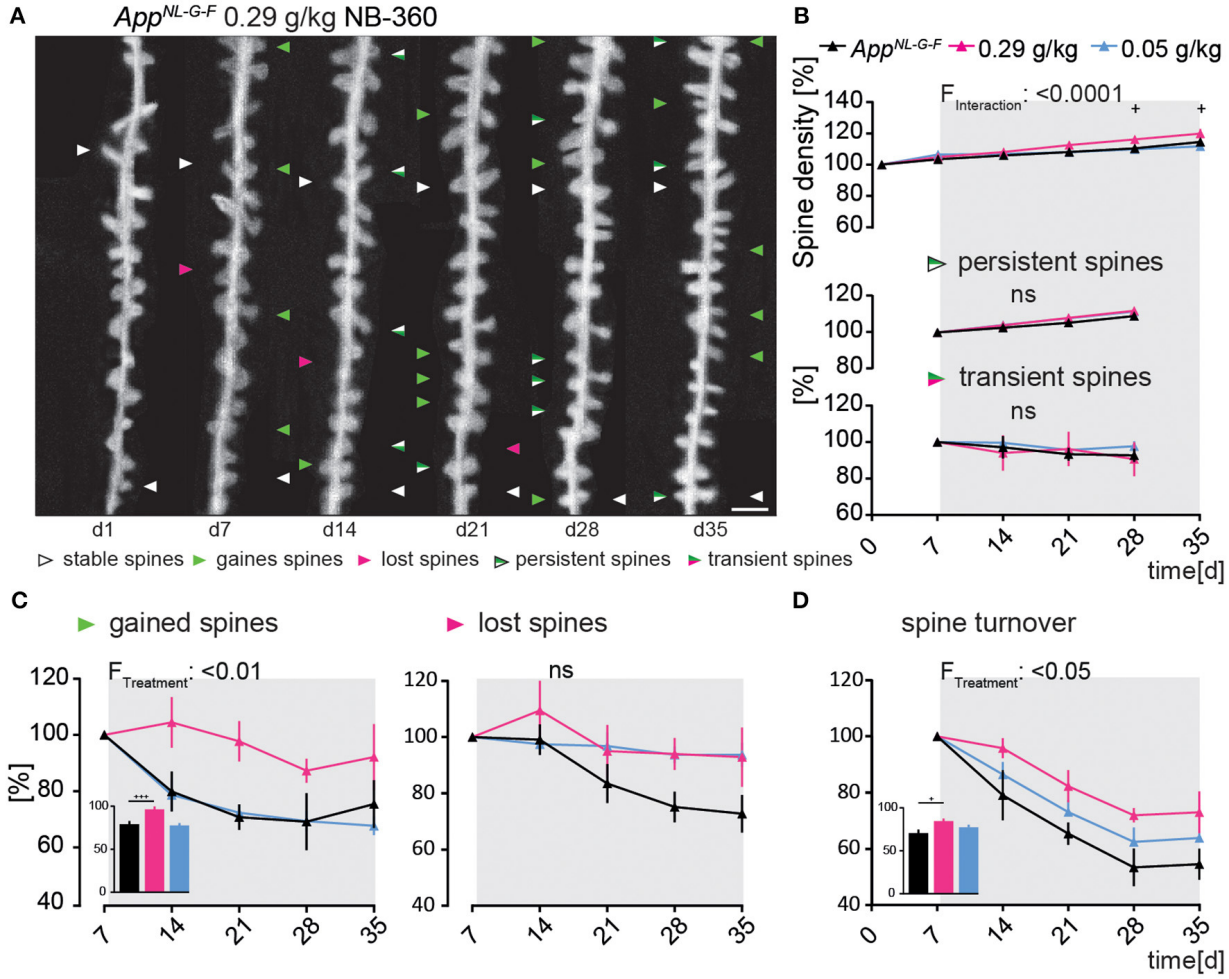
Preventive BACE1-inhibition using high-dosed NB-360 specifically enhanced the formation of new dendritic spines In the *App*^*NL*−*G*−*F*^ mouse model. **(A)** Representative *in vivo* two-photon recordings of eGFP-labeled basal tuft dendrites in the hippocampal CA1 *stratum oriens* layer of *App*^*NL*−*G*−*F*^GFP-M mice treated with 0.29 g/kg NB-360 for four consecutive weeks starting at 4 months of age. Arrowheads mark representative spines that were stable (white), newly formed (green), or lost (magenta). Gained spines that did not stabilize (green/magenta, present < 7 days) were defined as transient, whereas gained spines that did stabilize were defined as persistent (green/white, present > 7 days). Scale bar: 2 μm. **(B,C)** BACE1-inhibitor intervention had a significant effect on spine density distant to Aβ-deposits in *App*^*NL*−*G*−*F*^GFP-M mice (*p*_TW_ < 0.0001), mainly due to a change in the formation of new spines (*p*_TW_ = 0.0098). High-dosed NB-360 treatment increased new spine formation compared with vehicle and low-dose NB-360 treatments (*p*_OW_ < 0.0001). The fractions of persistent, transient, and lost spines were not significantly changed by the treatments (*p* > 0.05). **(D)** BACE1-inhibition had a significant effect on spine turnover rate (*p*_TW_ = 0.0154), while the high-dose treatment group also reached significance according to one-way-ANOVA testing of differences between the treatment groups (*p*_OW_ = 0.0162). *N* = 6 animals per group, *n* = 7–10 dendrites per animal. Data are presented as mean ± SEM. Bonferroni *post-hoc* test results: ^+^*p*_OW_ < 0.05, ^+++^*p*_OW_ < 0.001, *p*_TW_ from two-way ANOVA and *p*_OW_ from one-way ANOVA.

*App*^*NL*−*G*−*F*^GFP-M mice treated with the high dose of NB-360 showed a significant increase in spine density compared to untreated animals [*F*_(10, 75)_ = 4.47; *p* ≤ 0.0001, Figure 3B], mainly due to an enhanced formation of new spines (Figure 3C). The fraction of newly gained spines increased by 17% compared to the vehicle group [*F*_(2, 15)_ = 6.389; *p* = 0.0098, Figure 3C]. The high dose of NB-360 caused a non-significant increase in the fraction of lost spines [*F*_(8, 15)_ = 2.679; *p* = 0.1012, Figure 3C]. Due to the change in spine dynamics, the spine turnover ratio was also significantly elevated in high-dosed NB-360 treated *App*^*NL*−*G*−*F*^GFP-M mice [*F*_(2, 15)_ = 5.585; *p* = 0.0154, Figure 3D], although there was no effect on the fractions of transient or persistent spines (Figure 3B).

*Ex vivo* immunostainings showed that the area covered by oligomeric Aβ stained with NAB228 decreased significantly under high-dosed NB-360 treatment compared to vehicle-treated animals (565.6 ± 69.07 μM^3^ vs. 1,464 ± 128.4 μM^3^; *p* < 0.0001, Supplementary Figure 1C). The effect of high-dosed NB-360 treatment on the area covered by fibrillary Aβ stained with FSB was not as pronounced as on the oligomeric Aβ-plaque components but still significant (15.65 ± 4.14 μM^3^ vs. 146.7 ± 31.67 μM^3^; *p* = 0.0002, Supplementary Figure 1C). Frequency distribution analysis also showed that under high-dosed NB-360 treatment, NAB228-positive Aβ-plaques generally occupied a smaller area of the brain volume evaluated than Aβ-plaques of untreated mice (Supplementary Figure 1D).

NB-360 in a low dose (0.05 g/kg) did not affect spine plasticity in *App*^*NL*−*G*−*F*^GFP-M *in vivo* (Figures 3A–D).

### BACE1-inhibition affects hippocampal spine dynamics and density in *App^*wt*^*GFP-M mice in a dose-dependent manner

In order to compare our present results with previous studies (Filser et al., 2015; Zhu et al., 2016; Blume et al., 2018), we also tested the effect of BACE1 inhibition on hippocampal dendritic spine density in *App*^*wt*^GFP-M mice.

**Figure 4:**
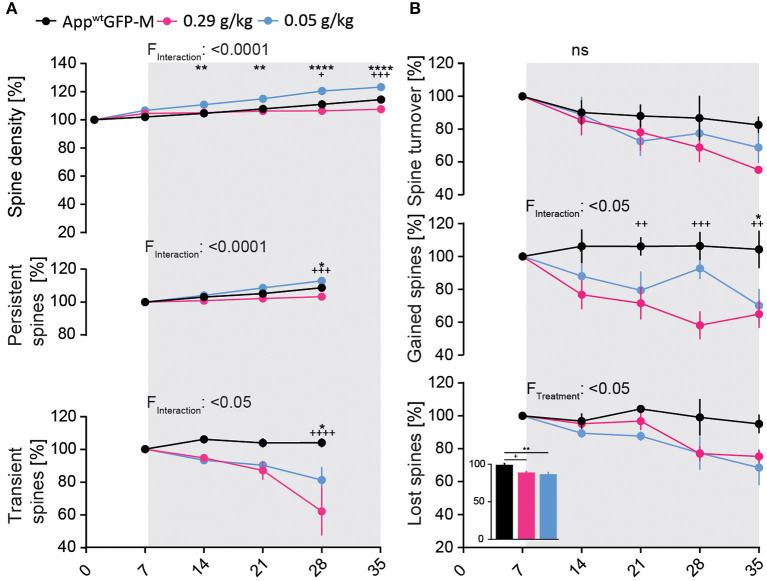
BACE1-inhibition affects spine kinetics and density in *App*^*wt*^*GFP-M* mice of hippocampal dendrites in a dose-dependent manner. **(A)** A significant decrease in spine density was observed in *App*^*wt*^GFP-M mice with high-dose NB-360 treatment (0.29 g/kg), while a significant increase was observed with low-dose NB-360 treatment (0.05 g/kg) (*p*_TW_ < 0.0001). This effect is reflected in the dose-dependent treatment effect on the fraction of persistent spines (*p*_TW_ < 0.0001) as well as on the fraction of transient spines (*p*_TW_ = 0.0172). **(B)** NB-360 treatment at high and low doses significantly decreased the formation of new spines (*p*_TW_ = 0.0132) as well as the fraction of lost spines (*p*_TW_ = 0.0356). This effect was more pronounced with low-dosed NB-360 treatment than in high-dose and vehicle treatment groups (*p*_OW_ = 0.0039). The effects on spine turnover rate did not reach significance. *N* = 6 animals per group, *n* = 7–10 dendrites per animal. Data are presented as mean ± SEM. Bonferroni *post-hoc* test results: *^/+^*p*_TW/OW_ < 0.05, **^/++^*p*_TW/OW_ < 0.01, ^+++^*p*_TW/OW_ < 0.001, ****^/++++^*p*_TW/OW_ < 0.0001, *p*_TW_ from two-way ANOVA and *p*_OW_ from one-way ANOVA.

We observed a significant 7% decrease in total spine density after 4 weeks of treatment in the high-dosed NB-360 treatment group. Surprisingly, treatment with the low-dose of NB-360 (0.05 g/kg) resulted in a significant 9% increase in total spine density at the last day of treatment compared to vehicle group [*F*_(10, 65)_ = 11.05; *p* ≤ 0.0001; Figure 4A]. This was unexpected, as we observed a significant decrease in the formation of new spines over the imaging period with both BACE1-inhibitor doses compared to vehicle group [*F*_(8, 52)_ = 2.744; *p* = 0.0132; Figure 4B].

Both NB-360 doses significantly decreased the fraction of lost spines [*F*_(2, 13)_ = 4.359; *p* = 0.0356, Figure 4B], but only the low-dose NB-360 treatment increased the density of persistent spines [*F*_(6, 39)_ = 7.643; *p* ≤ 0.0001; Figure 4A]. Furthermore, we found a notable decrease in the fraction of transient spines at both NB-360 dosages [*F*_(6, 39)_ = 2.977; *p* = 0.0172; Figure 4A].

## Discussion

After the unfortunate failure of previous clinical trials with BACE inhibitors, the hypothesis emerged that Aβ-directed treatment approaches in patients with Alzheimer would be most efficient when they start in the pre-symptomatic phase of the disease, before irreversible neuronal and synaptic damage has occurred (Moussa-Pacha et al., 2019; Hampel et al., 2020). In addition, a recent study revealed that BACE1-inhibition almost halted plaque formation in APP-transgenic mice, especially distant to pre-existing plaques, whereas plaque growth was only moderately reduced (Peters et al., 2018). In contrast to the dense-cored Aβ-plaques, which are typically observed in transgenic AD mouse models and consist mainly of Aβ-fibrils (Sasaguri et al., 2017), the Aβ-deposits in APP knock-in mouse mice consist predominantly of loosely bound prefibrillar Aβ-oligomers (Sacher et al., 2019), which are similar in their composition to human Aβ-deposits in early stage of Alzheimer's disease (Saido et al., 1995; Sasaguri et al., 2017).

In this study, we showed that the structural spine plasticity of CA1 pyramidal cells is impaired even when dendrites were located distant to Aβ-deposits. The reduction in spine dynamics potentially causes impairment of synaptic function and could be the structural correlate of altered long-term potentiation (LTP) in the APP knock-in mouse model. Previous studies have shown that LTP is impaired in the prefrontal cortex of APP knock-in mice as early as 3–4 months of age and in the hippocampus by latest 6–8 months of age (Latif-Hernandez et al., 2020). In 10-month-old animals, a significant impairment of memory as well as transfer performance was demonstrated (Sacher et al., 2019). Hippocampus-dependent spatial memory performance is also impaired in older APP knock-in mice (Masuda et al., 2016; Sakakibara et al., 2019). The memory deficits in older APP knock-in mice may be attributed to the early impairment of LTP as well as the associated reduction in spine plasticity, which was also observed in this study.

LTP induced by high-frequency excitation of pre-synapses promotes the persistence of existing spines (Heine et al., 2008) as well as the formation of new spines (Engert and Bonhoeffer, 1999; Nägerl et al., 2004) and thus results in an overall strengthening of the synaptic connection between pre- and post-synapses (Matsuzaki et al., 2004; Okamoto et al., 2004). Soluble Aβ-oligomers are known to disrupt the induction of LTP and provoke long-term depression (LDP) (Cullen et al., 1997; Hu et al., 2008; Klyubin et al., 2014). However, although Aβ-oligomers significantly impaired synaptic function in the present APP knock-in mouse model at 5 months of age, as reflected in the decrease in structural plasticity of dendritic spines, we saw no loss of post-synapses, as it was shown in later stages of AD (Ferrer and Gullotta, 1990; Moolman et al., 2004; Spires et al., 2005; Koffie et al., 2009).

For this reason and in accordance with the previous literature, the APP knock-in mouse line is an attractive model to investigate preventive therapeutic approaches such as BACE1-inhibition, with the aim to inhibit Aβ42-generation and aggregation, thus reducing their toxicity on pre- and post-synapses.

We tested the dose dependence of BACE1-intervention on structural synaptic plasticity in APP knock-in mice, administering NB-360 at a high dose (0.29 g/kg) as well as a low dose (0.05 g/kg). The choice of inhibitor doses was based on their strong efficacy in wild-type animals, with the low dose causing a 78% reduction in brain and plasma Aβ42-levels and the high dose (0.25 g/kg) achieving an 84% reduction of Aβ42-levels (Neumann et al., 2019). In previous preclinical mouse studies, NB-360-dosages of 0.25 g/kg for 21 days in wild-type animals as well as Sez6 knockout mice (Zhu et al., 2016); 0.25 g/kg for 90 days in APPPS1 mice and 0.25 mg/g for 49 days in APP23 mice (Keskin et al., 2017) were used and no overt changes in the health and weight of treated mice were observed. However, as in previous studies (Zhu et al., 2016; Peters et al., 2018), we observed a loss of fur pigmentation in APP knock-in mice after 10 to 14 days of treatment at both doses of NB-360. This effect is caused by the unspecific inhibition of BACE2 (Neumann et al., 2015, 2019; Shimshek et al., 2016), a protease involved in melanogenesis by processing the pigment cell-specific melanocyte protein (Rochin et al., 2013).

In this study, we showed that early treatment with a high dose of the BACE1-inhibitor NB-360 was able to interfere with Aβ-generation and could thereby counteract the impaired spine dynamics in the APP knock-in mouse model. We observed a significant increase in the formation of new dendritic spines resulting in net spine turnover rates on the last day of treatment that was comparable to those in untreated wild-type control animals (73 ± 7% vs. 82 ± 5%). Due to the increased formation of new spines, we also saw increased total spine density in APP knock-in mice receiving the high dose of NB-360, although there was no such effect with the low-dosed inhibitor.

In control mice (*App*^*wt*^GFP-M mice, further referred to as wild-type mice), the low-dose NB-360 treatment led to an increase in spine density. We believe that this is the consequence of a stabilization process of existing spines reflected in an increase in persistent spine density. However, high-dose NB-360-treatment in wild-type mice led to a significant decrease in total spine density, mostly due to reduced formation of new spines. These results are consistent with previous studies showing that BACE inhibition under physiological conditions induces structural and functional synaptic changes *via* disruption of SEZ6 function (Filser et al., 2015; Zhu et al., 2016; Blume et al., 2018). Indeed, Sez6 is exclusively cleaved by BACE1 and required for normal dendritic arborization as well as synaptic plasticity (Gunnersen et al., 2007; Pigoni et al., 2016). Moreover, physiological concentrations of Aβ-peptides have beneficial effects on synaptic plasticity (Puzzo et al., 2008). Thus, under physiological conditions, pharmacological suppression of Aβ-levels at synapses and inhibited processing of pre- and post-synaptic substrates of BACE1, especially Sez6, lead to the observed impairment of structural plasticity and dendritic spine density in wild-type mice.

In contrast, low-dosed NB-360 treatment might be neuroprotective by causing the accumulation of sAPP-α (Fol et al., 2016) as well as unprocessed APP (Klevanski et al., 2015) at synapses, thus outbalancing the synaptotoxic effects and positively influencing the stabilization of synapses and spines. Thereby, this mechanism of spine stabilization might have led to the observed significant increase in the density of persistent spines in wild-type mice treated with low-dose NB-360. It is also conceivable that the low-dosed NB-360 treatment did not yet downregulate SEZ6 processing to the extent that synaptic plasticity was affected. In the APP knock-in mouse model it is conceivable, that the toxic effects of high Aβ-load on synaptic plasticity probably predominate to such an extent that when BACE1 is pharmacologically inhibited, the associated reduced processing of Sez6 shows no evident effect. However, it would be prudent to continuously measure soluble Sez6 in CSF during pharmacological intervention with BACE inhibitors to ensure that the synaptic function of BACE1 is upheld maintained over time.

In conclusion, our findings demonstrate that early pharmacological intervention with a BACE1-inhibitor could halt the pathological cascade leading to the loss of spines and neurons in the APP knock-in mouse model of AD by enhancing the structural plasticity of dendritic spines in the hippocampal CA1 *stratum oriens* layer. The relevance of our findings for the human disease remains to be established.

## Conclusion

In conclusion, *App*^*NL*−*G*−*F*^ mice showed a reduced formation of new spines, but the overall spine density was not yet affected, reminiscent of an early stage of AD. Our findings showed that BACE1-inhibition with NB-360 increased dendritic spine turnover rate by improving the formation of new spines in *App*^*NL*−*G*−*F*^ mice and thus counteracted the further progression of dendritic spine loss. After the failure of promising BACE1-inhibitors in clinical trials, it must be considered that cognitive function in symptomatic patients may not be rescuable, as the accumulation of Aβ and thus the degradation of synapses and neurons has proceeded too far at the onset of treatment. If administered at a very early disease stage prior to the substantial accumulation of the Aβ-protein and loss of synapses, therapeutic intervention with BACE1-inhibition may well arrest the pathology progression and spare the patient from severe cognitive impairment. Therefore, an individually tailored and primary preventive BACE1-inhibitor intervention may represent a promising therapeutic approach for AD.

## Data availability statement

The raw data supporting the conclusions of this article will be made available by the authors, without undue reservation.

## Ethics statement

All animal procedures followed a protocol approved by the local authorities (Regierung von Oberbayern, TVA-AZ: 55.2-1-54-2532-214-2016).

## Author contributions

TB performed all the measurements after establishing the hippocampal window surgery in the lab, analyzed, quantified the data, and wrote the manuscript. UN and DS contributed the BACE1 inhibitor. TS and TCS contributed the mouse model *App*^*NL*−*G*−*F*^. TB, SF, CS, FP, MB, and JH interpreted the data. TB, SF, and JH contributed to the conception and design of the study. CS and SF helped correcting the manuscript. All authors approved the final manuscript.

## Conflict of interest

UN and DS are employees of Novartis Pharma AG. SF and FP were employed by Evotec SE. The remaining authors declare that the research was conducted in the absence of any commercial or financial relationships that could be construed as a potential conflict of interest.

## Publisher's note

All claims expressed in this article are solely those of the authors and do not necessarily represent those of their affiliated organizations, or those of the publisher, the editors and the reviewers. Any product that may be evaluated in this article, or claim that may be made by its manufacturer, is not guaranteed or endorsed by the publisher.
