# Supplementary material for: β-secretase inhibition prevents structural spine plasticity deficits in AppNL-G-F mice
Source: Front Aging Neurosci. 2022 Jul 22;14:909586. doi: 10.3389/fnagi.2022.909586 (PMC9354544; doi:10.3389/fnagi.2022.909586)
Supplement: Supplementary file 1 [file Data_Sheet_1.docx]

Supplementary Material

1. **Supplemental methods**
   1. **Details on immunohistochemistry to quantify amyloid plaque load**

Hippocampal brain slices of APP*^NL-G-F^* mice treated with high-dosed NB-360 (0,29 g/kg) for four consecutive weeks stained against fibrillar Aβ with FSB and oligomeric Aβ with NAB228. We acquired three-dimensional 16-bit data stacks of 2048 × 2048 × 120 pixels from up to five different positions in the hippocampal CA1 *stratum oriens* layer at a lateral resolution of 0.17 μm/pixel and an axial resolution of 0.4 μm/pixel. Only Aβ-Plaques which were fully present in the slice were analysed. The laser power (NAB228: 633 nm and FBS: 405 nm) was not changed in between the acquisition of all Aβ-Plaques. To quantify the covered area by oligomeric as well as fibrillary Aβ, we utilized custom-written algorithms in Matlab (MathWorks, Natick, USA). The detailed method was described previously (Peters et al., 2018). GraphPad Prism was used to do statistical testing using the nonparametric Mann-Whitney U-test.

1. **Supplemental figure**

**
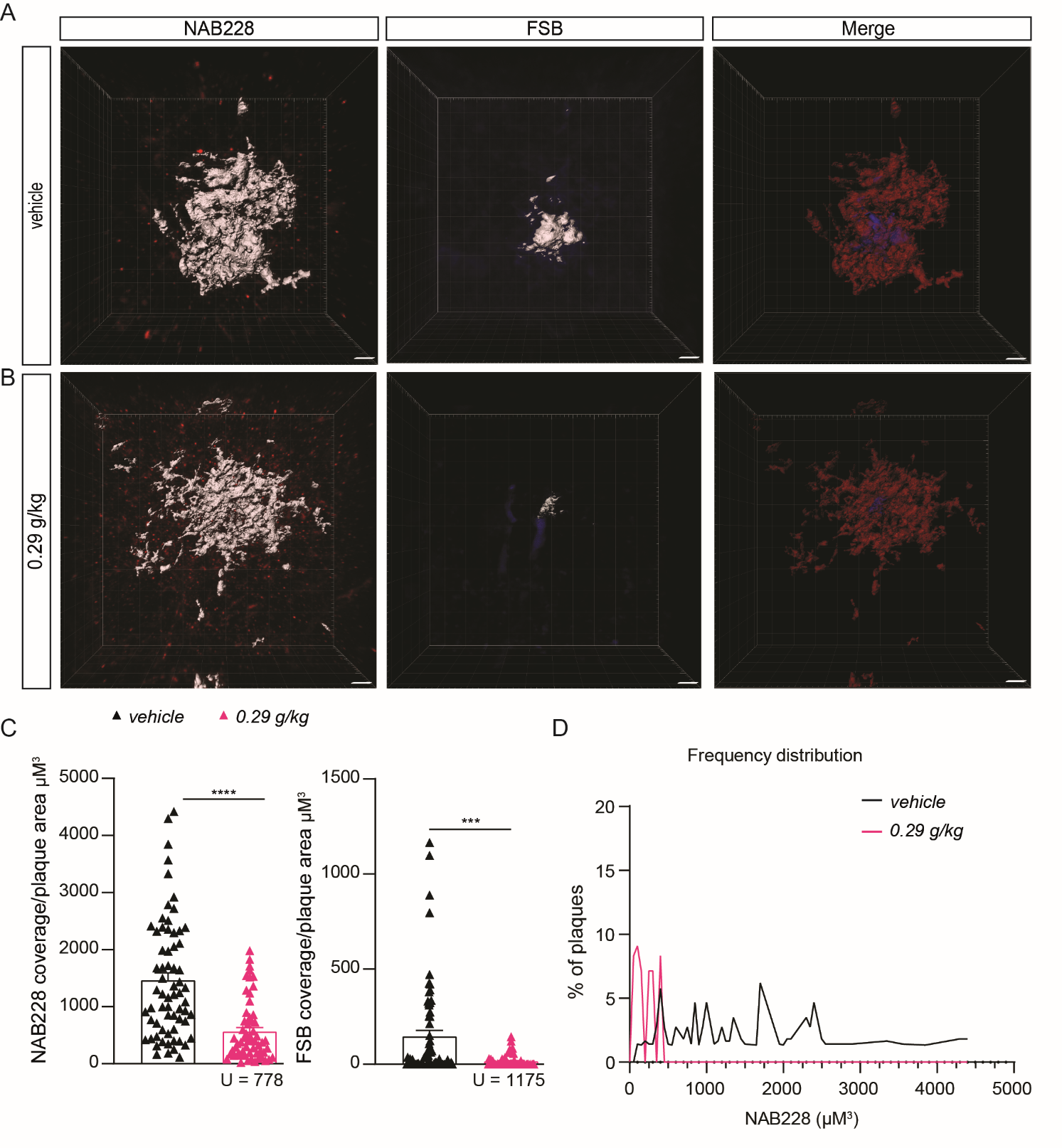
**

**Supplemental figure 1. Effect of high-dosed NB-360 treatment on hippocampal Aβ-plaque load in APP-NL-G-F mice.** Staining of oligomeric Aβ (NAB228, magenta) and fibrillary Aβ (FSB, cyan) in A) vehicle and B) high-dosed NAB-360 treated *App^NL-G-F^* mice. C) The plaque area covered by NAB228 staining was significantly decreased under high-dosed NAB-360 treatment compared to vehicle treatment (565.6 ± 69.07 μM^3^ vs. 1464 ± 128.4 μM^3^; p < 0.0001). The plaque area covered by FSB staining was also significantly lower in high-dosed NAB-360 treated *App^NL-G-F^* mice (15.65 ± 4.14 μM^3^ vs. 146.7 ± 31.67 μM^3^; p = 0.0002). D) The percentage of plaques per size group are shown with the oligomeric Aβ signal. N = 5 mice, n = 12-15 plaques per animal. For each graph, the U-value is listed. Data are presented as mean ± SEM. Mann-Whitney U-Test results: *** p < 0.0001, **** p < 0.00001.

REFERENCES

Peters, F., Salihoglu, H., Rodrigues, E., Herzog, E., Blume, T., Filser, S., et al. (2018). BACE1 inhibition more effectively suppresses initiation than progression of β-amyloid pathology. *Acta Neuropathol* 135, 695–710. doi: 10.1007/s00401-017-1804-9
